# Protocol of the VICTORIA study: personalized vitamin D supplementation for reducing or preventing fatigue and enhancing quality of life of patients with colorectal tumor - randomized intervention trial

**DOI:** 10.1186/s12885-020-07219-z

**Published:** 2020-08-08

**Authors:** Ben Schöttker, Sabine Kuznia, Dana Clarissa Laetsch, David Czock, Annette Kopp-Schneider, Reiner Caspari, Hermann Brenner

**Affiliations:** 1 Division of Clinical Epidemiology and Aging Research, German Cancer Research Center (DKFZ), Im Neuenheimer Feld 581, 69120 Heidelberg, Germany; 2 Network Aging Research, University of Heidelberg, Bergheimer Straße 20, 69115 Heidelberg, Germany; 3 Department of Clinical Pharmacology and Pharmacoepidemiology, University Hospital Heidelberg, Im Neuenheimer Feld 410, 69120 Heidelberg, Germany; 4 Division of Biostatistics, German Cancer Research Center (DKFZ), Im Neuenheimer Feld 581, 69120 Heidelberg, Germany; 5 Rehabilitation Clinic Niederrhein, Hochstraße 13-19, 53474 Bad Neuenahr-Ahrweiler, Germany; 6 Division of Preventive Oncology, German Cancer Research Center (DKFZ) and National Center for Tumor Diseases (NCT), Im Neuenheimer Feld 460, 69120 Heidelberg, Germany; 7 German Cancer Consortium (DKTK), German Cancer Research Center (DKFZ), Im Neuenheimer Feld 280, 69120 Heidelberg, Germany

**Keywords:** Randomized controlled trial, Vitamin D, Colorectal cancer, Rehabilitation, Fatigue, Quality of life, Depression, Infection, Inflammation, HbA_1c_

## Abstract

**Background:**

Cancer-related fatigue represents one major cause of reduced quality of life in cancer patients and can seriously affect the physical, emotional, and cognitive functioning impeding coping with the disease. Options for effective treatment of cancer-related fatigue are limited, consisting only of non-pharmacologic interventions like physical activity, psychosocial, and mind-body interventions. Recent evidence suggests that vitamin D_3_ supplementation might alleviate cancer-related fatigue. However, confirmation in a randomized controlled trial is needed.

**Methods:**

In this multicenter, randomized, double-blind, placebo-controlled trial, 456 colorectal cancer (CRC) patients aged 18 years and older are being recruited in three German rehabilitation clinics. Study inclusion requires hospitalization of at least 3 weeks at such a clinic, a diagnosis of non-metastatic CRC (stage I-III), surgical removal of the tumor within the past 9 months, and season-adapted vitamin D insufficiency or deficiency. Eligible patients are randomly assigned to a personalized regimen of vitamin D_3_ or placebo for 12 weeks. In the intervention group, a loading dose of 20,000 or 40,000 IU vitamin D_3_ will be administered daily during the first 11 days, followed by a maintenance dose of 2000 IU daily. Patients will complete questionnaires for secondary outcomes (fatigue subdomains, quality of life and subdomains, depression, functional well-being, and infection frequency). Blood and urine samples will be collected for analyses of safety parameters (hypervitaminosis D, hypercalcemia, hypercalciuria, and renal impairment) and efficacy biomarkers (25-hydroxyvitamin D, HbA_1c_, white blood cell count, leukocyte subtype counts, serum C-reactive protein, uric acid, creatinine, triglycerides, total, low- and high-density lipoprotein cholesterol).

**Discussion:**

This trial tests whether a personalized vitamin D_3_ dosing regimen reduces or prevents fatigue among non-metastatic CRC patients by treating the underlying vitamin D deficiency/insufficiency. If efficacy can be confirmed, personalized vitamin D_3_ supplementation could be used as a tertiary prevention measure in addition to non-pharmacological treatments of cancer-related fatigue in CRC patients. We expect to detect an effect of vitamin D_3_ supplementation on secondary outcomes like quality of life, depression, functional well-being, infections, inflammatory biomarkers, diabetes mellitus, and dyslipidemia.

**Trial registration:**

European Clinical Trials Database: EudraCT-No: 2019–000502-30, January 21, 2019; German Clinical Trials Register (DRKS): DRKS00019907, April 30, 2019.

## Background

Colorectal cancer (CRC, ICD-10 C18–21) accounts for more than 60,000 new cases and more than 25,000 deaths per year in Germany [1]. The prognosis has particularly improved for earlier stages of the disease [2]. However, detriments in the Quality of Life (QoL) often persist [3].

One common cause of reduced QoL in CRC patients is cancer-related fatigue. Multiple observational studies have consistently shown that approximately one-third of CRC survivors suffer from fatigue, not only shortly after diagnosis and initial treatment, but also in the long run [3–5]. Fatigue can be highly distressing for the patients: it negatively affects their physical, emotional, and cognitive functioning, and limits their ability to cope with activities of daily living and work [4–6].

So far, only non-pharmacologic approaches, such as physical activity, psychosocial, and mind-body interventions, were found to be effective in alleviating cancer-associated fatigue [7]. A promising pharmacological approach could be to normalize the vitamin D status in CRC patients with vitamin D deficiency. 25-hydroxyvitamin D (25(OH)D) is the best-established biomarker of vitamin D status and its serum levels are typically very low in CRC patients among all stages [8]. Vitamin D deficiency (25(OH)D levels < 30 nmol/L) was detected among 59% of 2912 CRC patients from a large German study during or shortly after first-line treatment and was strongly associated with poor survival [9]. Results from several observational studies also found associations between low 25(OH)D levels and fatigue, exhaustion, and frailty, both in cancer patients and in the elderly population [10, 11].

To date, few controlled trials have addressed the hypothesis of a beneficial effect of vitamin D_3_ supplementation with respect to fatigue, and they mostly suffered from substantial methodological limitations. A pilot study by Kerley et al. observed reduced fatigue in the vitamin D_3_ group but improvement was not statistically significantly higher than in the placebo group [12]. Of note, the trial included only 19 patients with obstructive sleep apnea [12]. A trial by Khan et al. among 60 women with breast cancer observed no statistically significant effect of vitamin D_3_ supplementation on change in fatigue scores over 5 weeks (*p* = 0.15) but was limited by lack of randomization and adequate statistical power [13].

Recently, two randomized placebo-controlled vitamin D trials in general population samples with the endpoint fatigue were published. The trial by Nowak et al. included 120 subjects with hypovitaminosis D (25(OH)D levels < 50 nmol/L) and fatigue who were randomized to either one-time 100,000 IU vitamin D_3_ or placebo [14]. A statistically significant (*p* = 0.01) reduction of the fatigue score was observed 4 weeks after the intervention, which correlated with the rise in the serum 25(OH)D levels. In a smaller trial by Witham et al., 50 individuals were randomized to 100,000 IU vitamin D_3_ or placebo every 2 months for 6 months but no effect was found [15]. However, the trial did not exclude subjects with normal 25(OH)D levels (> 50 nmol/L) and the mean baseline 25(OH)D level was 46 nmol/L. Since supplementation appears to be most beneficial to people with vitamin D deficiency [16], it may not be surprising that supplementation did not show a statistically significant effect in this small, apparently healthy trial population.

The randomized, placebo-controlled, double-blind VICTORIA trial will test the efficacy of vitamin D_3_ supplementation over 12 weeks on fatigue in non-metastatic CRC patients with low vitamin D status. The proposed trial will be by far the largest to address the efficacy of vitamin D_3_ supplementation on fatigue as a primary outcome and the first to be conducted in non-metastatic CRC patients in whom the prevalence of vitamin D insufficiency/deficiency and fatigue are particularly high. It will employ a novel approach using a personalized vitamin D_3_ loading dose and will have adequate power to detect a clinically relevant reduction in fatigue.

### **Objectives**

The primary objective of this trial is to test whether a personalized vitamin D_3_ dosing regimen reduces or prevents fatigue (primary outcome) among stage I-III CRC patients with season-adapted vitamin D insufficiency or deficiency.

The secondary objectives are to assess the efficacy of the treatment on sub-domains of fatigue (physical, emotional and cognitive), QoL (overall and domain-specific), probable depression, functional well-being (FWB), infection frequencies, and various biomarkers (25(OH)D, HbA_1c_, white blood cell count, leukocyte subtype counts, serum c-reactive protein (CRP), uric acid, creatinine, triglycerides, total, low-density lipoprotein (LDL) and high-density lipoprotein (HDL) cholesterol). The study further aims to evaluate the safety of the treatment regarding the occurrence of hypervitaminosis D, hypercalcemia, hypercalciuria, and renal dysfunction.

## Methods/Design

### **Study design**

The trial is conducted as a national multicenter, randomized, double-blind, and placebo-controlled phase III trial using a confirmatory approach. By applying a parallel group design, the participants are randomly assigned in a 1:1 ratio to either the intervention group or to the control group. Recruitment is carried out in three large German rehabilitation clinics and is assumed to take approximately 24 months. The list of trial sites can be obtained from the German Clinical Trials Register (DRKS00019907).

The trial protocol and this manuscript have been developed in line with the Standard Protocol Items: Recommendations for Interventional Trials (SPIRIT) guideline [17].

### **Outcomes and endpoints**

The trial outcomes have been chosen in order to determine the efficacy and the safety of the intervention.

The primary outcome **fatigue** will be evaluated by using the **Functional Assessment of Chronic Illness Therapy-Fatigue (FACIT-F) fatigue subscale**, version 4.0, a commonly used and well-validated measure of fatigue in people with cancer and other chronic health conditions [18]. The tool assesses self-reported tiredness, weakness, and difficulty conducting common activities due to fatigue. Higher scores represent less fatigue. The primary endpoint will be measured as the mean difference in FACIT-F fatigue subscale between intervention and placebo group at trial week 13–16. A mean difference of ≥3 FACIT-F fatigue subscale points will be considered a clinically relevant difference [19]. Additionally, the mean difference in change of FACIT-F fatigue subscale from baseline to trial week 13–16 between intervention and placebo group will be determined as a secondary endpoint.

**Subdomains of fatigue** will be evaluated using the **European Organization for Research and Treatment of Cancer – Cancer related Fatigue (EORTC QLQ-FA12) questionnaire.** This is an alternative to the FACIT-F fatigue subscale without being a global fatigue assessment tool [20, 21], with higher scores representing more severe fatigue. Items which are relevant for the VICTORIA study include physical, emotional and cognitive fatigue domains. They will be assessed as the mean differences in EORTC QLQ-FA12 physical, emotional and cognitive fatigue scores between intervention and placebo group at trial week 13–16 as well as mean differences in changes in these scores from baseline to trial week 13–16 between intervention and placebo group.

**Quality of life** will be determined with the **European Organization for Research and Treatment of Cancer – Core Quality of life questionnaire with 30 items (EORTC-QLQ-C30),** version 3.0. The questionnaire is used to gauge the overall and domain-specific QoL in cancer patients [22]. Higher scores represent better functioning. Items which are relevant for the VICTORIA study include the five functional scales (assessing physical, role, emotional, cognitive, and social functioning) and one global health status/QoL scale. Endpoints are the mean differences in overall and domain specific quality of life scores of the EORTC QLQ-C30 questionnaire between intervention and placebo group at trial week 13–16 as well as mean differences in changes in these scores from baseline to trial week 13–16 between intervention and placebo group. Mean differences ≥5 points in the overall and domain specific scores of the EORTC QLQ-C30 will be considered clinically relevant differences [23].

**Probable depression** will be ascertained with the **Geriatric depression scale (GDS-15)** which has been developed for use among older adults [24, 25]. The focus on the elderly is crucial for this trial as the mean age of the CRC patients to be included is expected to be approximately 65 years. An overall score ≥ 5 points is considered as probable depression [25]. Endpoint will be the mean difference in GDS-15 scale between intervention and placebo group at trial week 13–16 as well as the mean difference in changes in this scale from baseline to trial week 13–16 between intervention and placebo group.

The **FACIT-F FWB subscale** will be used to assess the **FWB** by querying about limitations in the ability to work, coping with the disease, and general life satisfaction [18]. Higher scores represent better well-being. Endpoint will be the mean difference in the FACIT-F FWB score between intervention and placebo group at trial week 13–16 as well as the mean difference in changes in this score from baseline to trial week 13–16 between intervention and placebo group.

**Infection frequency** will be assessed by a self-developed questionnaire. Participants will be asked to state the number of infection episodes for the following infections during the last 12 weeks: Infections of the upper and lower respiratory tract, gastrointestinal infection with diarrhea, cystitis, and fever higher than 38 °C. The total infection frequency will be the sum of all reported infection episodes. If this sum is lower than the number of stated fever episodes with ≥38 °C, the latter will be used as the total infection frequency. Endpoints will be the mean differences in infection frequencies (total, upper respiratory, and lower respiratory tract infections) between intervention and placebo group at trial week 13–16.

Finally, serum und urine parameters will be measured at every sample collection to evaluate the following secondary endpoints:

- Mean difference in levels of total serum 25(OH)D between intervention and placebo group at trial day 12–21 and in trial week 13–16 as well as mean difference in change of levels of total serum 25(OH)D from baseline to trial day 12–21 and from baseline to trial week 13–16.
- Mean serum 25(OH)D levels > 50 nmol/L in intervention group at trial day 12–21 and in trial week 13–16.
- Mean differences in levels of biomarkers (white blood cell count (WBC), leukocyte subtype counts (band neutrophils, segmented neutrophils, eosinophils, basophils, lymphocytes, and monocytes), serum CRP, serum uric acid, serum creatinine, serum total cholesterol, serum LDL cholesterol, serum HDL cholesterol, and serum triglycerides between intervention and placebo group at trial day 12–21 and in trial week 13–16 as well as mean difference in change of levels of these biomarkers from baseline to trial day 12–21 and from baseline to trial week 13–16.
- Mean difference in HbA_1c_ levels between intervention and placebo group at trial week 13–16 as well as mean difference in change of HbA_1c_ levels from baseline to trial week 13–16.
- Differences in frequency of safety parameters (including hypervitaminosis D, hypercalcemia, hypercalciuria, and renal dysfunction (estimated glomerular filtration rate (eGFR) < 30 ml/min/1.73 m^2^) between intervention and placebo group at trial day 12–21 and in trial week 13–16.
- Mean differences in levels of safety parameters (including albumin-corrected serum calcium, urine calcium/creatinine ratio, and eGFR) between intervention and placebo group at trial day 12–21 and in trial week 13–16.

### **Study procedures**

#### Study population

Inclusion and exclusion criteria are listed in Table 1. As there will be no preferences regarding age or sex of the patients to be included and since most non-metastatic CRC patients in Germany undergo in-patient rehabilitation within several weeks after first-line treatment, the approach will ensure high representativeness of this population. Recruitment during rehabilitation was chosen because patients are being admitted as inpatients, usually for 3 weeks, allowing administration of the loading dose under medical supervision to ensure close observation for adverse events.

**Table 1:** Inclusion and exclusion criteria

Inclusion Criteria	Exclusion Criteria
• Age ≥ 18 years • Non-metastatic CRC patients (UICC stage I-III) • Within 9 months after surgical removal of tumor • At least 3 weeks in-patient rehabilitation in a cooperating clinic is planned • Sufficient knowledge of the German language and mental capabilities to be able to give written informed consent and comply with the study requirements	• No season adapted vitamin D insufficiency or deficiency (see Table 2 for definition) • BMI > 40 kg/m^2^ • Severe anemia (Hemoglobin < 8.0 g/dl) • Severe renal impairment (eGFR < 30 ml/min/1,73 m^2^ calculated with the CKD-EPI equation [26]) • Hypercalcemia (Albumin-corrected serum calcium > 2.65 mmol/L) • Hypercalciuria (Random urine calcium ≥0.60 mmol/mmol creatinine) • High-dose vitamin D_3_ therapy (≥ 2000 IU daily or similar dosage which corresponds to ≥2000 IU daily) • Therapy with vitamin D analogs • Topical therapy with vitamin D_3_ or vitamin D analogs • Therapy with high-dose calcium supplements (> 1000 mg calcium daily) • Therapy with cardiac glycosides • Hypersensitivity towards ingredients in Dekristol® 20,000/1000 I.E.: peanuts, soy, gelatin, lactose, maize starch or sucrose or ingredients in the placebo capsules (mannitol, silicon dioxide) • Nephrolithiasis with symptoms in the last 12 months • Pseudohyperparathyreodism • Sarcoidosis • Participation in another interventional trial • Pregnancy, planned pregnancy in the next 12 weeks, or lactation • No use of adequate contraceptive measures in women with childbearing potential

Abbreviations: *BMI* body mass index; *CKD-EPI* Chronic Kidney Disease Epidemiology Collaboration; *eGRF* estimated glomerular filtration rate; *UICC* Union for International Cancer Control

##### Definition of season-adapted vitamin D insufficiency

Only patients with season-adapted vitamin D insufficiency will be included. This means that the 25(OH)D level cut-off will depend on the month of recruitment. The rationale of season-adapted cut-offs is that only patients having vitamin D insufficiency for the entire year should be included. This is a challenge because people with low 25(OH)D levels in winter usually have a sufficing level in summer. The calculation of the season-adapted cut-offs for vitamin D insufficiency is shown in Table 2. The median 25(OH)D levels by calendar month were derived from the ESTHER study, a population-based prospective cohort study with 25(OH)D measurements for 9585 study participants who were recruited over 2 years in Saarland, a small state with approximately 1 million inhabitants located in south-west Germany [27]. The highest median 25(OH)D level was observed in August with 55 nmol/L. Using the threshold for vitamin D insufficiency of the US American Institute of Medicine [28], the cut-off for vitamin D insufficiency in this month was set to 50 nmol/L. The season-adapted cut-offs in the other calendar months were calculated by subtracting their median difference to August 25(OH)D levels from 50 nmol/L using the following equation:

**Table 2:** Season-adapted cut-offs for vitamin D insufficiency by calendar month

Calendar month	Median 25(OH)D [nmol/L]	Difference to median of August [nmol/L]	Season-adapted 25(OH)D cut-off [nmol/L]
January	40	15	35
February	40	15	35
March	40	15	35
April	40	15	35
May	41	14	36
June	50	5	45
July	53	2	48
August	55	0	50
September	53	2	48
October	52	3	47
November	45	10	40
December	43	12	38

Season-adapted 25(OH)D level cut-off month X = 50 nmol/L – (55 nmol/L - median 25(OH)D level month X).

Thus, the cut-offs reflect the natural seasonal variation of the 25(OH)D level of a hypothetical person in Germany with a 25(OH)D level of 50 nmol/L in August, the month during which the study participants had the highest 25(OH)D serum levels. An advantage of using season-adapted cut-offs is a constant recruitment rate of patients with vitamin D insufficiency over the whole year. Otherwise, the majority of the participants would be recruited in winter and only few in summer.

### Intervention

For the verum, 20,000 IU (Dekristol® 20,000 I.E., Mibe GmbH Arzneimittel) or 2000 IU colecalciferol (two tablets Dekristol® 1000 I.E., Mibe Arzneimittel GmbH) are encapsulated and filled up with a mixture of mannitol and colloidal silicon dioxide (Füllstoff DAC). The placebo consists solely of the hard gelatine capsules with filler. To ensure blinding, verum and placebo capsules are indistinguishable regarding appearance, quantity, weight, and packaging.

Patients, who have consented to participate in the trial, are randomly allocated to either vitamin D_3_ or placebo for a total of 12 weeks (84 days).

#### *Intervention arm*

The vitamin D_3_ treatment consists of a personalized loading dose, followed by a daily maintenance dose of 2000 IU. Several clinical trials have highlighted that 25(OH)D levels, achieved by vitamin D_3_ intake, strongly depend on a person’s 25(OH)D level and weight at baseline [29–31]. In order to provide the trial participants with a sufficient vitamin D_3_ dose, an equation to calculate individual loading doses is applied. The equation includes the baseline 25(OH)D level and body mass index (BMI) [31]:

Loading dose = 165 * BMI [kg/m^2^] * (70 – baseline 25(OH)D level [nmol/L]).

The equation by Jansen et al. was developed to increase 25(OH)D levels to 80 nmol/L, which is slightly above the generally accepted optimal level of 75 nmol/L [32]. In order to avoid non-physiological high doses of vitamin D_3_, the loading dose will be distributed in daily doses of 20,000 IU, which resembles the maximum amount of vitamin D_3_ that can be naturally produced in the skin by sunbathing in a swimsuit on a summer day [33]. The reasons for this are not safety concerns but mechanistic considerations regarding the vitamin D_3_ metabolism: Unbound vitamin D_3_ in the serum is of major importance for effective vitamin D activity [34, 35]. Its bioavailability in turn is higher when doses are given on a daily basis while avoiding single high doses [34, 35].

However, if the total personalized vitamin D_3_ loading dose of a participant is exceptionally high, 2 × 20,000 IU/day will be administered at the beginning to ensure that the entire vitamin D_3_ loading dose is administered within the first 11 days, while the patient is still in the rehabilitation clinic (for safety reasons). Accordingly, the maximum achievable loading dose of vitamin D_3_ is fixed at 420,000 IU over 11 days. This dose would only be necessary for an individual with a 25(OH)D level of 5 nmol/L (lowest expected baseline level) and a BMI of 40 kg/m^2^ (maximum possible BMI in this trial since patients with BMI > 40 kg/m^2^ are excluded). After administrating the last loading dose capsule, patients will continue with the maintenance dose on the next day until the end of the intervention (day 84). The daily maintenance dose (2000 IU) corresponds to the upper limit of the Endocrine Society’s recommendations for adults at high risk for vitamin D deficiency. It was chosen on the assumption that early stage CRC patients who only recently underwent rehabilitation have a higher turnover of vitamin D metabolites than individuals from the general population [32]. Depending on the loading dose, overall doses will range between 168,000 and 566,000 IU vitamin D_3_ taken over 84 days.

#### *Control arm*

Patients will receive placebo in the same schedule as in the intervention arm.

#### *Compliance*

To ensure a high adherence to the trial protocol, all patients are thoroughly instructed orally at regular visits and in writing via the patient information documents. Daily intake of study medication and potential adverse events (AEs) are recorded in the patient diary. The participant can agree to receive a weekly SMS, reminding them to take their medication and fill in the diary. It will be allowed to take forgotten maintenance doses of the week on the day of the SMS reminder.

The investigator documents the supply of the trial medication as well as the return of unused loading dose capsules and/or empty packages. The coordinating center (Division of Clinical Epidemiology and Aging Research at the German Cancer Research Center, Heidelberg) keeps records of the return of unused maintenance dose capsules and/or empty packages. Intake of less than 80% of the trial medication will be defined as incompliance.

#### *Concomitant medication*

The treatment of accompanying illnesses is only accepted if they are not subject to the exclusion criteria and if they are not expected to affect the trial’s outcome measures or to interfere with the trial medication. All additional treatments taken by the participants at inclusion or at any time during the trial are considered concomitant treatments and will be queried in the questionnaires. The following concomitant medication is strictly prohibited: vitamin D and vitamin D analogs, cardiac glycosides, and high-dose calcium supplements (> 1000 mg calcium daily).

#### *Dose modification*

Dosage adjustments are not permitted during the trial. In the case of a diagnosed vitamin D_3_ overdose, the treatment will be stopped immediately.

#### *Randomization and blinding*

The pharmacy of the Heidelberg University Hospital manages randomization by assigning each medication package a unique three-digit number according to a randomization list. The list is created using the randomization software “RITA – Randomization in Treatment Arms” (Version 1.31) before the actual manufacturing of the study medication and is kept sealed at the coordinating center until completion of the trial. Thus, trial physicians, medical staff, patients, and staff of the coordinating center including biometricians and scientists are blinded throughout the trial. The drug packages will be sent to the trial sites in blocks of six with equal numbers of verum and placebo packages to ensure block randomization. Stratification by study center will be ensured. Trial physicians will assign the intervention blindly by taking the medication package next in row from the open block. In addition to the trial medication, the study sites will receive a sealed envelope for each randomization number with information on whether the corresponding drug package comprised verum or placebo. The envelopes will be kept at the respective study centers and remain closed, except in case of emergency unblinding due to safety reasons (e.g. safety parameters meet stopping criteria). While the trial is ongoing, the clinical monitor will check the presence and intactness of the envelopes regularly. After completion of the clinical trial, intentional unblinding is performed by combining the documentation at the trial sites with the information of the pharmacy’s randomization list.

For safety reasons, multiple laboratory tests have to be conducted in the course of the trial. In order to avoid accidental unblinding of the study staff at the trial sites and the coordinating center by knowledge of a patient’s serum 25(OH)D, serum calcium or urinary calcium levels, pseudonymized laboratory test results will be sent to a medical documentarist at the coordinating center who has no access to patient-identifying data or further pseudonymized research data collected within the trial. The medical documentarist will check the safety parameters and will inform the study sites if any of the stopping criteria are met.

### Study flow

**Fig. 1:**
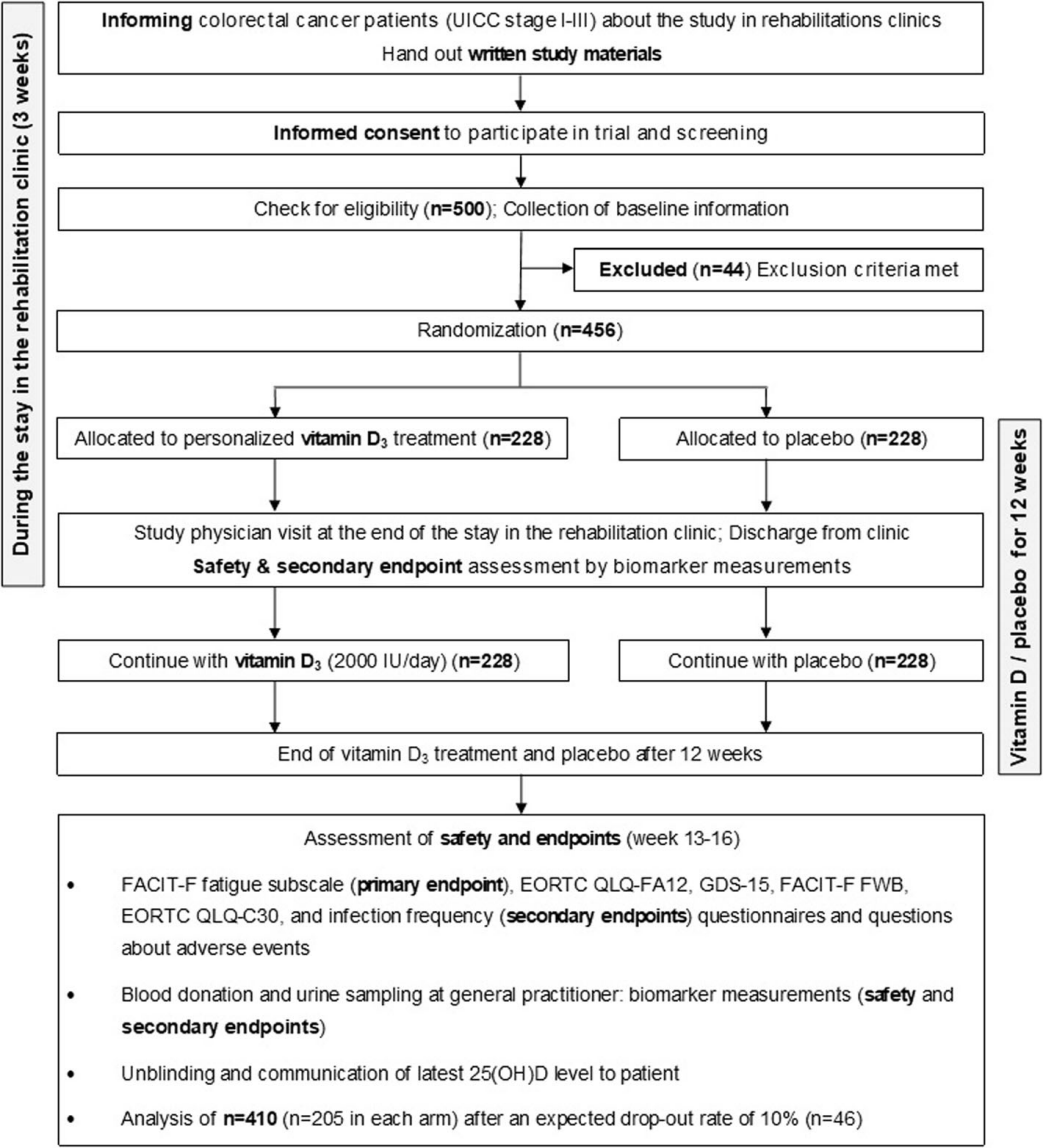
Overview of the study flow. Abbreviations: *EORTC QLQ-FA12* European Organization for Research and Treatment of Cancer – Cancer related Fatigue; *EORTC QLQ-C30* European Organization for Research and Treatment of Cancer – Core Quality of life questionnaire with 30 items; *FACIT-F* Functional Assessment of Chronic Illness Therapy-Fatigue; *FACIT-F FWB* Functional Assessment of Chronic Illness Therapy-Fatigue – Functional Well-being; *GDS-15* Geriatric Depression Scale with 15 items; *UICC* Union for International Cancer Control

The study flow is depicted in Fig. 1.

In total, 456 eligible CRC patients are being randomized in order to analyze 410 for the primary endpoint after an expected drop-out rate of 10%. The trial schedule is presented in Table 3.

**Table 3:** Detailed schedule of all observations to be performed during the clinical trial

Procedure	Screening Visit for eligibility	Visit 1 Start of treatment	Visit 2 End of rehabilitation	Collection of data via questionnaire/ phone and blood/urine sampling by GP
Rehabilitation clinic	Admission	–	Discharge	–
Time-point of data assessment	–	Day 1	Day 12–21	Week 13–16
Planned doses of vitamin D_3_ per day	–	Individualized initial dose (20,000 or 40,000 IU, followed by 2000 IU per day) for day 1–11	Maintenance dose of 2000 IU per day for day 12–21	–
Inclusion/exclusion criteria	●	●		
Written informed consent	●			
Randomization		●		
Trial medication intake		●	●	
Subject diary regarding days of drug administration		●	●	
Physical examination (weight, height, waist circumference)	●			
Urinary pregnancy test for women of childbearing potential	●			
Assessment of CRC treatment	●			●
Concomitant diseases / Medical history	●			
Concomitant medication	●			●
Query about concomitant use of vitamin D_3_ or vitamin D analogs in self-medication in addition to trial medication				●
Fatigue	●			●
Functional well-being, QoL, infection frequency, and depression	●			●
Blood sampling for biomarker efficacy and safety endpoints	●		●	●
Urine sampling for safety endpoints	●		●	●
(S)AE recording and protocol deviations		●	●	●

Abbreviations: *CRC* colorectal cancer; *GP* general practitioner; *QoL* Quality of life; *SAE* Serious adverse event

During a first informative appointment, patients are briefed about the study and receive the printed study information. After having enough time to read the information materials, the potential study participants have the opportunity to ask questions before the official screening visit commences.

#### *Screening*

The trial physician will conduct the informed consent discussion with eligible patients and obtain the written informed consent. For each patient who agreed to participate in the trial, the coordinating center will generate a unique 4-digit patient identification number, which is being documented along with the date of enrollment in the patient identification list. The waist circumference of the patient will be measured. In order to calculate the BMI, the height and weight will be gauged additionally. Women of child bearing potential have to undergo a urine pregnancy test. All patients will be asked to complete a study questionnaire as well as to provide a blood and urine sample to gather baseline information.

#### *Visit 1 (start of the treatment phase, day 1)*

After checking the in- and exclusion criteria, eligible patients will be included in the clinical trial. The trial physician will hand out the trial medication to the participant for the first 28 days. This includes the entire quantity of capsules for the calculated loading dose and the amount of the daily maintenance dose that supplies the patient until visit 2 and one additional week to prevent any interruptions of the intervention. Along with the trial medication, participants will receive a patient diary in which the intake of medication and potential AEs during the upcoming treatment period of 12 weeks are recorded.

#### *Visit 2 (day 12–21)*

At the end of the stay in the rehabilitation clinic, the participants will have completed the intake of the loading dose. To check their compliance, the patients will be asked to bring the empty trial medication packages of the loading dose and any leftover medication to this visit. A blood and urine sample will be collected to monitor biomarkers and safety parameters including hypervitaminosis D, hypercalcemia, hypercalciuria, and renal dysfunction. All suspected cases of AEs reported by the participants are assessed by the trial physician. Once it is confirmed that none of the safety parameters meet any of the criteria for discontinuation, the patient receives the remaining trial medication for the days 29 to 84. If any clinically relevant abnormalities are detected, the intake of the trial medication will be stopped immediately and further measures initiated, as appropriate.

#### *13 to 16 weeks after beginning of treatment*

After finishing administration of the trial medication by the end of week 12, the participants will be asked to fill out a questionnaire to assess fatigue symptoms and further outcomes, and to provide a final blood and urine sample at their general practitioner’s (GP’s) office. The filled-in questionnaire will be sent back to the coordinating center along with the patient diary, the unused trial medication and the empty medication packages.

The coordinating center assesses the compliance, checks the plausibility of the questionnaire replies, and records potential AEs that were noted for the outpatient period in the patient diary. The study physician evaluates each AE for severity and a potential causal relationship to the trial medication. He/she further takes measures to treat and follow-up AEs as necessary. Cases of hypercalcemia and hypercalciuria detected during the final laboratory test will be followed up for additional 2 months after the last intake of the trial medication to monitor normalization of the albumin-corrected serum calcium and the urine calcium to creatinine ratio.

After reporting the end of the trial of a participant, the participant will be immediately unblinded by receiving information from the coordinating center on the treatment, the results of the latest 25(OH)D measurement, and on options to treat vitamin D deficiency. The participant can decide in consultation with his/her GP whether to start (for the placebo group) or continue (for the verum group) vitamin D_3_ supplementation. Unblinding will be performed for each participant separately by opening a sealed envelope with the patient’s randomization number and the information if the corresponding drug package contained verum or placebo. The unblinding will be done at the latest 5 weeks after week 16. To ensure the integrity of the data, staff members of the coordinating center will have clearly defined roles and tasks, which are assigned via a delegation log.

In order to collect questionnaires at the end of the intervention from almost all trial participants, they will be contacted several times: first by a mailed questionnaire and ultimately by a phone call. Furthermore, they will get a monetary compensation (25 € if the questionnaire is sent back or answered by phone and a blood sample was provided).

### **Data management**

Data handling will be described in the trial data management plan. The Coordination Center for Clinical Trials, Heidelberg (KKS) provides and monitors a validated electronic case report form (eCRF) for remote data entry. The eCRF will be used for all data entry by study centers and the coordination center. All entries in the eCRF will be verifiable by source documents (if not explicitly exempt from this rule) and include:

- the randomization number (available once a patient is randomized),
- verification of compliance with in- and exclusion criteria,
- demographic data and data collected during the physical examination,
- concomitant diseases and medications,
- laboratory values,
- dates of participation in the study, of informed consent, and of dispensing study material and medication,
- AEs,
- date of drop-out and reasons for drop-out (voluntary information),
- and protocol deviations.

All patient data are documented pseudonymously. All documents and data pertaining to the trial will be stored at the study centers in the investigator site file (ISF) and at the coordinating center in the trial master file for at least 10 years after the end of the trial in accordance with the applicable regulations.

### Data collection and documentation by the trial sites

Written informed consent will be obtained for each participant before enrollment in the trial and will be kept in the ISF at the respective study centers. The investigators will enter the date and quantities of distributed and returned study medication as well as all participant data collected during rehabilitation into the eCRF, except biomarker results from the second blood and urine sample collection to avoid unblinding. All data entries in the eCRF will undergo an automatic online check for plausibility and consistency, which is defined in the data validation plan. In case of implausibility, a warning message will be produced during data entry. A responsible investigator or a designated representative will be obliged either to correct the implausible data or to confirm its authenticity and give an appropriate explanation. The responsible data manager will check all explanations and will resolve the warning if the explanation is appropriate.

The responsible investigator has to confirm the correctness of all entries in the eCRF with a dated electronic signature. The time points and frequency are pre-defined in the eCRF specification.

All missing data or inconsistencies will be reported back to the study centers and have to be clarified by the responsible investigator before database lock. If no further corrections are to be made in the database, it will be declared locked and can be used for statistical analyses.

### Data collection and documentation by the coordinating center

The coordinating center enters the pseudonymized laboratory results of the second and the final sample collection as well as study data from questionnaires and patient diaries in the eCRF. To ensure the integrity of the data, staff members of the coordinating center will have clearly defined roles and tasks which will be assigned via a delegation log. For example, coordinating center staff members who contact the study participants will not have access to the eCRF, the laboratory results or the sealed envelopes containing the information on the allocation of treatment. Staff members who open these envelopes or check the pseudonymized laboratory results of the study participants, in turn, will not have access to any patient-identifying data or other study data.

The data management will check the completeness, the validity, and the plausibility of the entered data using validation programs, which will generate queries where applicable. The head of the coordinating center or a designated representative will confirm the correctness of all entries in the eCRF by a dated signature as defined in the eCRF specification.

### Questionnaires

Each patient will complete a questionnaire in the rehabilitation clinic at baseline and in study weeks 13 to 16. The questionnaires will address lifestyle factors (smoking, alcohol consumption, diet, physical activity), CRC therapy (chemotherapy, radiotherapy, operation), and medical history (common diseases, family history of diseases) as well as the (validated) tools for assessment of the outcomes.

### Blood and urine sampling and laboratory measurements

Patients will be asked to provide a blood and a spontaneous urine sample upon screening (baseline), at the end of rehabilitation (day 12–21) and once again in the study weeks 13 to 16. The first two samples will be collected in the rehabilitation clinic (inpatient) while the final sample will be collected by the patient’s GP (outpatient). For the sampling at the GP’s office, the coordinating center will send pseudonymized sampling kits to the participants after trial week 12. One part of the samples will be used to determine biomarkers immediately (as efficacy or safety outcomes). Another part will be stored at the coordinating center for potential future post-hoc analyses with novel biomarkers.

All blood samples drawn during the trial will be either collected in clotting activator containing tubes to analyze serum or in EDTA tubes to analyze whole blood. The urine samples will be donated in a collection cup of 40 ml from which a volume of 10 ml is extracted using Urin-Monovette®, Luer, Germany. Any residual volume will be discarded. The quantity of collected blood and urine samples is shown in Table 4.

**Table 4:** Time and volume of sampling

Setting	No. of sample	Phase of clinical trial	Blood Sampling		Urine Sampling
			EDTA tube	Serum tube	
Inpatient	1	Screening	1 × 2.6 ml	1 × 7.5 ml	1 × 10 ml
	2	Day 12 to 21	1 × 2.6 ml	4 × 7.5 ml	2 × 10 ml
Outpatient	3	Week 13 to 16	1 × 2.6 ml	1 × 7.5 ml, 3 × 9 ml	2 × 10 ml

**1st and 2nd sample**: The EDTA tube, one serum tube, and 10 ml urine will be prepared for transportation by the laboratories with which the rehabilitation clinics normally cooperate. This procedure ensures rapid transport and analysis of biomarkers within 48 h. The remaining samples (if any) will be sent via express mail to the coordinating center for aliquoting and storage at − 80 °C.

**3rd sample**: The GP will send all samples to the laboratory of the coordinating center via express mail. The latter will immediately send the EDTA tube, one serum tube, and 10 ml urine via express mail to the same cooperating laboratories that also process the first two specimens. The remaining samples will be stored at the laboratory of the coordinating center at − 80 °C. By using the same laboratories for the sample analysis, bias originating from different measurement methods is avoided.

To comply with the pre-analytical requirements, it is ensured that the transport time from sampling to analysis is less than 48 h and the temperature of the samples during transport is between 2 and 25 °C [36]. Serum tubes are inverted 3–5 times immediately after withdrawal and then stored at room temperature for 30 to 60 min to allow blood clotting. Tubes are subsequently centrifuged at 2500–3500 rpm for 10 min. EDTA tubes will be inverted eight to 10 times to ensure an even mixture of EDTA and blood.

The laboratories cooperating with the rehabilitation clinics will measure all parameters with standard state-of-the-art lab methods.

### **Safety assessment**

Participants will be asked about concomitant medication and diseases before inclusion. To minimize the risk of AEs and exacerbation of present conditions, patients will only be included if conditions that are listed as contraindications in the Summary of Product Characteristics of colecalciferol 20,000 IU or require special safety monitoring are not present (see exclusion criteria, Table 1). In the patient information document, patients will be informed about potential risks associated with the trial participation and instructed to contact the trial physician in case of serious medical problems. The observation period of AEs will begin with the first administration of the trial medication and end with its last administration. Events happening before the first administration are defined as medical history. AEs will be queried during every visit with the responsible investigator and additionally within the patient diary during the entire intervention phase. All AEs will be documented in the eCRF stating the participant’s randomization number, the start and end date, a description, the intensity, the seriousness, the relationship with the study medication, the measures taken, and the outcome.

All serious adverse events (SAEs) must be reported by the investigator to the pharmacovigilance department of KKS using a standardized form within 24 h after initial observation or awareness of the event. Every SAE will be subject to a second assessment by a designated person, who will be independent of the reporting investigator. All SAEs and their relevance for the risk-benefit assessment of the trial as well as the final report will be evaluated continuously during the trial.

A steering committee will be convened to ensure the ethical conduct of the trial and protect the rights as well as the welfare of the patients. The board consists of the coordinating investigator, the head of the coordinating center, the deputy head of the coordinating center, and the clinical pharmacology consultant. By periodically assessing the safety of the intervention and reviewing potential safety issues, amendments to the further trial conduct (modification, continuation, closure) will be decided and documented.

### **Quality control and assurance**

Internal standard operating procedures will be followed for the preparation, implementation, documentation, and the analyses of the clinical trial. All applicable regulations will be followed. The pharmacy of the University Hospital Heidelberg is holding a manufacturing license and is therefore authorized to produce the required study medication. Packing, labeling, and blinding will take place according to the applicable GCP and Good Manufacturing Practice regulations and standards.

As required by the German Drug Law (AMG) for multicenter trials, the sponsor has appointed a coordinating investigator (German: Leiter der klinischen Prüfung). Moreover, every trial site has appointed one principal investigator and at least one deputy investigator. All trial physicians and trial centers comply with the qualification requirements of the responsible Ethics Committee in terms of professional education, experience in clinical trials and equipment. Since the trial medication is well characterized, of low risk, and tested in a non-critical indication (fatigue), a Data Monitoring Committee has not been set up [37].

All data obtained in the course of the trial will be treated pursuant to the German Federal Data Protection Act (BDSG) and the European ordinance (EU) 2016/679. The individual participants will be exclusively identified by their patient identification and randomization numbers. The investigators will provide direct access to source data/documents for trial-related monitoring, audits, and regulatory inspection. Each participant will have to consent via written informed consent to direct access to their original medical records for trial-related monitoring, audit, and regulatory inspection.

Qualified staff will regularly monitor every study site by reviewing source documents, entries into the eCRFs, and essential documents to ensure that the trial complies with the protocol and regulatory requirements. Monitoring includes an on-site initiation visit, regular on- and off-site visits during the recruitment phase and a close-out visit. Before the study start, the participating sites will be personally trained and introduced to all study specific procedures during the on-site initiation visits. After each visit, the monitor will prepare a report for the sponsor and a follow-up letter with findings and eventual necessary measures for the sites. All procedures will be pre-defined in the monitoring manual.

All planned substantial changes of the clinical trial need to be signed by the sponsor, the coordinating investigator, the biometrician and the clinical pharmacology consultant. According to §10 of the GCP ordinance (GCP-V), protocol amendments are submitted in writing to the responsible Ethics Committee and the national competent authority.

### **Statistical analysis plan**

#### Sample size estimation

The sample size calculation is based on the primary endpoint FACIT-F fatigue subscale. We assume that the FACIT-F fatigue subscale is approximately normally distributed. An increase in the FACIT-F fatigue subscale by three points was found to be a clinically relevant reduction of fatigue [19]. The assumed mean and standard deviation of the FACIT-F fatigue subscale are taken from the representative study of Jones et al. [4], which were 38.5 and 10.8, respectively. With a significance level of 0.05 and 80% power, 205 patients are needed in each group to detect a score difference of three or more points using a two-sample t-test for the mean difference, i.e. 410 patients are required in total. The number of patients to be randomized was calculated assuming a 10% drop-out rate. With these assumptions 500 CRC patients need to be screened and 456 eligible persons randomized to achieve the analyzable sample size of *n* = 410.

Given the annual number of overall 1400 eligible CRC patients reported by the participating three rehabilitation clinics in 2016, recruiting of 456 eligible stage I-III CRC patients should be feasible within 24 months.

### Statistical methods

All analyses will be done using SAS software version 9.4 or higher. Homogeneity of the treatment groups will be described by comparison of the demographic data and the baseline values of key variables. All statistical tests will have a two-sided significance level of 0.05. Besides, 95% confidence intervals will be estimated for all outcomes in the placebo and verum group.

The primary analysis will test the null hypothesis.

H_0_: The FACIT-F fatigue subscale at week 13–16 is the same in the two groups.

versus the alternative hypothesis.

H_1_: The FACIT-F fatigue subscale at week 13–16 is different in the two groups.

The primary endpoint FACIT-F fatigue subscale will be analyzed with an intention-to-treat approach. The intention-to-treat population will include all randomized study participants with data for the primary endpoint and who did not withdraw consent during the trial. A per-protocol analysis will be done additionally as a sensitivity analysis. The per-protocol population will exclude study participants who meet the following criteria:

- Fatigue questionnaire missing at trial week 13–16
- Error in timing of collection of fatigue questionnaire at trial week 13–16
- After enrollment, it becomes evident that patient met exclusion criteria at time of recruitment or did not meet inclusion criteria.
- Incompliance to trial medication (defined as intake of less than 80% of capsules) unless treatment was terminated due to safety reasons
- Self-reported intake of vitamin D or vitamin D analogs in addition to the trial medication.
- Criteria for discontinuation of study medication were met but study medication intake was continued.

We will test on normal distribution for the outcome FACIT-F fatigue subscale with the Shapiro-Wilks test. If normal distribution can be assumed, the primary test statistic will be the two-sample t-test for the mean difference. If normal distribution cannot be assumed for the FACIT-F fatigue subscale, we will log-transform the FACIT-F fatigue subscale and test for normal distribution once more with the Shapiro-Wilks test. If normal distribution can neither be assumed for FACIT-F fatigue subscale nor log-transformed FACIT-F fatigue subscale, a Wilcoxon Rank-Sum test will be applied. In addition, appropriate 95% confidence intervals will be estimated for the FACIT-F fatigue subscale in the two groups and for the difference in FACIT-F fatigue subscale between the two groups.

A priori defined subgroup analyses will be conducted for groups defined by age (< 65 / ≥ 65 years), sex (male / female), CRC stage (I or II / III), 25(OH)D levels at screening (< 30 / ≥ 30 nmol/L), season at screening (Dec-Feb/Mar-Mai/Jun-Aug/Sept-Nov), BMI at screening (< 30 / ≥ 30 kg/m^2^), FACIT-F fatigue subscale at screening (≤ 34 / > 34 points), mild to moderate anemia at screening (hemoglobin 8–10 mg/dl / > 10 mg/dl), chemotherapy and/or radiotherapy in 9 months before screening (yes / no), chemotherapy and/or radiotherapy during trial (yes / no), geriatric depression scale (GDS-15) score at follow-up (< 5 / ≥ 5 points), insomnia at follow-up (EORTC QLQ-C30 insomnia item < 3 / ≥ 3 points), pain at follow-up (EORTC QLQ-C30 pain scale < 6 / ≥ 6 points), use of strong opioids (ATC codes N02AB03, N02AA01, N02AG01, N02AA51, N02AA03, N02AG04, N02AA53, N02AA05, N02AJ18, N02AJ19, N02AA55, N02AA56, N02AJ17, N02AX06, N02AE01, and N02AA25) at follow-up (yes / no), use of psycholeptics (ATC code N05) at follow-up (yes / no), and use of corticosteroids for systemic use (ATC code H02) at follow-up (yes / no).

In addition, a subgroup analysis will be conducted for study participants, who most likely profit from the vitamin D_3_ intervention because the following conditions apply combined (yes / no): No protocol deviations (patients did not withdraw consent during the trial, were adherent to the trial medication (defined as taking at least 80% of all capsules), and did not take vitamin D_3_ or vitamin D analogs in addition to the trial medication), FACIT-F fatigue subscale ≤34 at screening, and neither use of strong opioids nor psycholeptics at follow-up.

A mean difference ≥ 3 FACIT-F fatigue subscale points will be considered a clinically relevant difference [19].

Depending on the availability of future funding for genotyping of all randomized trial participants, further subgroup analyses are planned that stratify trial participants by genetic susceptibility for low 25(OH)D levels or cancer related fatigue [38, 39].

As a sensitivity analysis, a linear regression model will be carried out with FACIT-F fatigue subscale as the dependent variable and treatment group as the independent variable. The linear regression model will be adjusted for all variables named in the subgroup analysis while using some variables continuously instead of categorically (age, hemoglobin levels at screening, 25(OH)D levels at screening, BMI at screening, FACIT-F fatigue subscale at screening, insomnia scale at follow-up and pain scale at follow-up). If appropriate, missing covariate values will be imputed with multiple imputation.

All secondary endpoints with a continuous scale will be analyzed with the same statistical methods as described for the primary endpoint. All dichotomous secondary endpoints will be tested with a Chi^2^ test. AEs will be analyzed with descriptive statistics including frequencies of SAEs.

Lastly, the success of the personalized vitamin D_3_ intervention in raising the serum 25(OH)D level will be evaluated in the intervention group at trial day 12–21. Furthermore, the success of the maintenance dose to maintain sufficient vitamin D status in the intervention group will be evaluated in trial week 13–16. To be successful, the mean 25(OH)D levels in the group with personalized vitamin D_3_ intervention should be higher than 50 nmol/L. This translates into the following test hypotheses:

- H_0_: Mean 25(OH)D_personalized intervention_ ≤ 50 nmol/L
- H_1_: Mean 25(OH)D_personalized intervention_ > 50 nmol/L

To test these hypotheses, we will conduct a one-sample t-test on the mean 25(OH)D levels at a one-sided significance level of 0.025. 25(OH)D levels may be log-transformed if this improves the approximation of a normal distribution (to be tested with a Shapiro-Wilks test).

*Exploratory sub-project: “Determinants of the achieved 25(OH)D levels by the personalized vitamin D*_*3*_*intervention”*

In the following, we describe the statistical methods of an observational sub-project for the secondary outcome “25(OH)D level”. This solely observational research project shall be addressed in those 228 VICTORIA trial participants who received the personalized vitamin D_3_ intervention and adhered to the trial medication (defined by taking at least 80% of the trial medication capsules), which will result in a final sample size *n* < 228. If 25(OH)D levels or log-transformed 25(OH)D levels are normally distributed (to be tested with a Shapiro-Wilks test), a linear regression model will be carried out with a continuous 25(OH)D level variable. In addition, a logistic regression model will be used with a dichotomized 25(OH)D level variable (< 50 nmol/L / ≥ 50 nmol/L) as the dependent variable.

Covariates for the linear and logistic regression model will be study center, age, sex, baseline 25(OH)D, compliance up to time of blood sampling, difference between exactly calculated and rounded supplied loading dose, number of days between last intake of trial medication and blood sampling, season at recruitment, baseline BMI, baseline waist circumference, baseline smoking, baseline physical activity, baseline physical functioning (EORTC QLQ-C30 subscale), baseline Charlson Comorbidity index, baseline frailty, baseline FACIT-F fatigue subscale, GDS-15 total score ≥ 5 points (at baseline), baseline anxiety (GAD-7 score), baseline pain (EORTC QLQ-C30 pain scale), CRC stage, time since CRC tumor surgery, stoma, concomitant use of vitamin D products (at follow-up), chemotherapy and/or radiotherapy in 9 months before trial (yes / no), chemotherapy and/or radiotherapy during trial (yes / no), concomitant use of laxatives (at baseline and/or follow-up), concomitant use of other drugs limiting vitamin D_3_ bioavailability at baseline and/or follow-up (phenytoin, barbiturates, systemic glucocorticoids, rifampicin, isoniazid, cholestyramine, orlistat, dactinomycin or systemic azole-antimycotics), nutritional vitamin D intake (at follow-up), appetite loss (at baseline and/or follow-up), sun exposure in last summer before baseline, skin type, solarium use in last 2 months before baseline, frequency of diarrhea (at baseline and/or follow-up), vitamin D binding protein levels and single nucleotide polymorphisms (SNPs) previously shown to be associated with low 25(OH)D levels. If appropriate, missing covariate values will be imputed with multiple imputation.

Mean 25(OH)D levels and proportions of study participants with 25(OH)D levels < 50 nmol/L will be presented distinctly for factors that were statistically significant (*p* < 0.05) determinants of achieved 25(OH)D levels.

The analyses will be carried out both for the 25(OH)D measurement from day 12–21 (end of loading dose consumption) and for the 25(OH)D measurement from week 13–16 (end of maintenance dose consumption).

#### *Interim analyses*

No interim analyses are planned for the primary outcome FACIT-F fatigue subscale in trial week 13–16 and the following ones have no implications for the ongoing of the trial.

Interim analyses are planned with the first half of the intended final sample size to be randomized, i.e. 228 study participants with available blood samples from week 13–16. The analyses will be focused on 25(OH)D-related secondary outcomes, all safety parameters and for the exploratory sub-project: “Determinants of the achieved 25(OH)D levels by the personalized vitamin D_3_ intervention”.

## Discussion

The present trial is by far the largest to address the efficacy of vitamin D_3_ supplementation on fatigue as a primary outcome and the first to be conducted in non-metastatic CRC patients in whom the prevalence of vitamin D insufficiency/deficiency and fatigue are particularly high [4]. Its novel methodological aspects are the use of season-adapted serum 25(OH)D cut-offs to determine suitability for inclusion in the trial and a personalized vitamin D_3_ loading dose over several days, thereby avoiding a single non-physiological high dose. The use of placebo has been considered to be ethically justifiable in CRC patients where vitamin D insufficiency is commonly found but usually neither diagnosed nor treated. Participants in the intervention group might benefit from the treatment of their vitamin D deficiency by prevention or reduction of fatigue symptoms. Daily administered oral vitamin D_3_ treatment in the proposed doses is considered acceptable for CRC patients concerning the expected benefit-risk profile.

The results of the trial are highly relevant for practice: Firstly, the trial population is representative of non-metastatic CRC patients suffering from vitamin D insufficiency/deficiency and attending a German rehabilitation clinic. The in−/exclusion criteria are kept to a necessary minimum to ensure generalizability. Secondly, vitamin D_3_ is not patented and available on the market at low cost. Consequently, the trial could be used as a base for a policy change offering a cost-effective screening and treatment of vitamin D deficiency to CRC patients in rehabilitation clinics. This would not only have a major impact on the therapy of fatigue and QoL but possibly on the survival of this population as well [40].

The choices of the trial intervention and the trial end-points have been discussed with the *Deutsche ILCO e.V.* (a patient advocacy group for CRC). We were assured that fatigue is a highly patient-relevant outcome for CRC patients within months to years after curative treatment and represents an unmet need, which complies with reports in the scientific literature [41, 42].

### **Trial status**

At the time of submission, recruitment for the VICTORIA study is planned to start in summer 2020.

## Supplementary information

**Additional file 1.** List of authorities involved in the approval of the trial.

## Data Availability

The data will not be published to an Open Access Platform. After completion of the study, interested scientists can request data use and receive pseudonymized data upon approval of this application by the sponsor.

## Abbreviations

25(OH)D: 25-hydroxyvitamin D
AE: Adverse event
AMG: German drug law (Deutsches Arzneimittelgesetz)
ATC: Anatomical-Therapeutic-Chemical Index
BDSG: Federal Data Protection Act (Bundesdatenschutzgesetz)
BfArM: Federal Institute for Drugs and Medical Devices (Bundesinstitut für Arzneimittel und Medizinprodukte)
BMI: Body mass index
CRC: Colorectal cancer
eCRF: Electronic Case Report Form
EORTC QLQ-C30: European Organization for Research and Treatment of Cancer – Core Quality of life questionnaire with 30 items
EORTC QLQ-FA12: European Organization for Research and Treatment of Cancer – Cancer related Fatigue
FACIT-F: Functional Assessment of Chronic Illness Therapy-Fatigue
FWB: Functional well-being
GDS-15: Geriatric depression scale (GDS-15)
GCP-V: Good Clinical Practice Ordinance (GCP-Verordnung)
GP: General Practitioner
ICH: International Council on Harmonization of Technical Requirements for Registration of Pharmaceuticals for Human Use
ICH-GCP: ICH harmonized tripartite guideline on GCP
ISF: Investigator Site File
KKS: Coordination Center for Clinical Trials (Koordinierungszentrum für Klinische Studien Heidelberg)
QoL: Quality of life
SAE: Serious Adverse Event
UICC: Union for International Cancer Control
