# Supplementary material for: Protocol of the VICTORIA study: personalized vitamin D supplementation for reducing or preventing fatigue and enhancing quality of life of patients with colorectal tumor - randomized intervention trial
Source: BMC Cancer. 2020 Aug 8;20:739. doi: 10.1186/s12885-020-07219-z (PMC7414549; doi:10.1186/s12885-020-07219-z)
Supplement: Supplementary file 1 — Additional file 1. List of authorities involved in the approval of the trial. [file 12885_2020_7219_MOESM1_ESM.pdf]

Additional File 1

List of authorities involved in the approval of the trial.

Competent Ethics Committee ("Federführende Ethikkommission"):

- State Chamber of Medicine in Rheinland-Palatinate (Ethikkommission der Landesärztekammer Rheinland-Pfalz), Deutschhausplatz 3, 55116 Mainz

Local Ethics Committee ("Beteiligte Ethikkommission"):

- Chamber of Medicine Westfalen-Lippe (Ethikkommission der Ärztekammer Westfalen-Lippe), Gartenstr. 210 – 214, 48147 Münster

National Competent Authority for the approval of clinical drug trials in Germany:

- The Federal Institute for Drugs and Medical Devices (Bundesinstitut für Arzneimittel und Medizinprodukte, BfArM), Kurt-Georg-Kiesinger-Allee 3, 53175 Bonn
